# Wildlife Warning Signs: Public Assessment of Components, Placement and Designs to Optimise Driver Response

**DOI:** 10.3390/ani3041142

**Published:** 2013-12-17

**Authors:** Amy R. F. Bond, Darryl N. Jones

**Affiliations:** Environmental Futures Centre, Griffith University, Nathan, Qld 4111, Australia; E-Mail: d.jones@griffith.edu.au

**Keywords:** wildlife–vehicle collisions, road signs, sign design, driver behaviour, mitigation, road ecology, urban wildlife

## Abstract

**Simple Summary:**

Wildlife warning signs are aimed at reducing wildlife–vehicle collisions but there is little evidence that they are effective. Improving these sign designs to increase driver response may reduce wildlife–vehicle collisions. We examined drivers’ responses to different wildlife warning sign designs through a public survey. The presences of some sign components and sign position were assessed. Drivers’ responses to eight graphically displayed signs and animal- and vehicle-activated signs were ranked and participants indicated the sign to which they were most likely to respond. Three signs ranked highly. Animal- and vehicle-activated signs were also ranked highly by participants. More research into optimising wildlife warning sign designs is needed.

**Abstract:**

Wildlife warning signs are the most commonly used and widespread form of road impact mitigation, aimed at reducing the incidence of wildlife–vehicle collisions. Evidence of the effectiveness of currently used signs is rare and often indicates minimal change in driver behaviour. Improving the design of these signs to increase the likelihood of appropriate driver response has the potential to reduce the incidence of wildlife–vehicle collisions. This study aimed to examine and assess the opinions of drivers on wildlife warning sign designs through a public opinion survey. Three currently used sign designs and five alternative sign designs were compared in the survey. A total of 134 drivers were surveyed. The presence of temporal specifications and an updated count of road-killed animals on wildlife warning signs were assessed, as well as the position of the sign. Drivers’ responses to the eight signs were scaled separately at three speed limits and participants indicated the sign to which they were most likely to respond. Three signs consistently ranked high. The messages conveyed by these signs and their prominent features were explored. Animal-activated and vehicle speed-activated signs were ranked very highly by participants. Extensive field trials of various sign designs are needed to further this research into optimizing wildlife warning sign designs.

## 1. Introduction

Wildlife–human interactions can occur frequently in urban environments, particularly at the front of urban sprawl expansion [1]. One such interaction that is particularly conspicuous is the wildlife–vehicle collisions that often occur in urban areas, especially where habitat remnants are still present in the landscape or the urbanised edge is expanding into rural or natural landscapes [2]. In these peri-urban areas, higher human densities are often coupled with higher traffic volumes and road densities, which increase the likelihood of wildlife coming into contact with roads and vehicles, and therefore the risk of wildlife–vehicle collisions [2]. This can be seen in a state-wide spatial analysis of wildlife–vehicle collisions in New South Wales (NSW) that revealed several hotspots concentrated around urban centres, such as Canberra, Newcastle and Byron Bay [3]. 

High incidence of wildlife–vehicle collisions can have significant impacts: increasing wildlife population mortality, influencing population demographics if there are age or sex biases in road mortality, reducing wildlife dispersal success, reducing gene flow between populations, animal welfare issues, causing human injury and occasionally fatalities, and the financial costs to society associated with human injury, vehicle damage, wildlife rehabilitation (for those that survive), and removal of animal carcasses [4,5]. Mitigating these impacts of wildlife–vehicle collisions is, therefore, very important in urban areas, for both the wildlife and the humans involved. Additionally, societies where wildlife–vehicle collisions are reduced will benefit from the reduced financial burden of such interactions [4].

Wildlife warning signs are the most commonly used and widespread form of road mitigation [5,6]. These signs are aimed at reducing the incidence of wildlife–vehicle collisions, and therefore reducing injuries and fatalities to wildlife and drivers, as well as vehicle damage [6]. Despite their common use, however, evidence of their effectiveness is inconsistent; most often, sign effectiveness has not been evaluated [7,8]. For example, a lighted, animated deer crossing sign on State Highway 82 in Colorado, USA was reported to reduce vehicle speeds, but this was minimal and, hence, ineffective at reducing the deer-vehicle collision rate [9]. In contrast, temporary flashing deer warning signs reduced deer-vehicle collisions by 51% during deer migrations and reduced the number of vehicles recorded to be speeding by at least 8km/h, but this effect did not last to the second year of the trial [10].

Al-Ghamdi and AlGadhi [11] compared seven camel warning signs of different designs and size, using the reduction in vehicle speed at night in response to the sign as the measure of effectiveness. The assessed signs included the standard warning sign design, both with and without diamond reflective material, and an alternative sign that included the words “camel-crossing” and an advisory speed; the three sign designs were also tested at various sizes [11]. Two of the signs did not elicit a reduction in vehicle speed, with the other signs significantly reducing speed by between 1.93 km/h and 6.51 km/h. Both sign designs were deemed effective at producing a relatively small, yet statistically significant, reduction in vehicle speed, with the larger signs that used diamond reflective material being the most effective [11]. However, when a series of similar large signs for moose-vehicle collisions were installed in conjunction with a public awareness campaign, records indicated a 41% drop in collisions with urban moose in Prince George, British Colombia, Canada [12].

Dique and colleagues [13] trialled differential speed wildlife warning signs that aimed to reduce koala-vehicle collisions during the breeding season. Although the number of incidents detected on trial roads was less than on control roads, there was no reduction of incidents during the trial periods when compared to the control periods [13]. In a recent trial of 16 wildlife (koala) warning signs with vehicle-activated flashing lights, speed reductions ranging from 0.49 km/h to 8.33 km/h were recorded when the vehicle-activated lights were turned on [14]. The vehicle speed at which the flashing lights were activated was altered experimentally, with vehicle speeds being consistently lower when lights were activated at 19 km/h, the lowest recommended speed for the radar units. Unfortunately, however, vehicle speeds before and after the signs were not recorded, and thus speeds were only compared with those when the signs were covered and no information on koala-vehicle collisions was available at the time of the study at the study sites [14].

Despite the limited evidence of their effectiveness, wildlife warning signs are likely to continue to be the most commonly implemented mitigation measure of wildlife–vehicle collisions due to their relatively low cost [4]. Therefore, improving the potential effectiveness of this inexpensive option may aid in reducing the impacts of wildlife–vehicle collisions. In Australia, as well as improving motorist safety [4], limiting these impacts may be particularly important where road mortalities to wildlife contribute to local population declines, if the landscape is unsuitable for other mitigation options, or the funds for more effective mitigation are not available. 

In Australia, the most commonly used wildlife warning signs are static, and very little research has been conducted on alternative sign designs and the potential to improve driver response. One exception was a study of an alternative wildlife warning sign designed and installed in Coles Bay and Bruny Island in Tasmania [15]. This design included an image of a car hitting a kangaroo, an advisory speed limit and the words “DUSK TO DAWN”. This sign was designed by the Tasmanian Wildlife Roadkill Collective that was made up of representatives from the Tasmanian Environment Centre, the University of Tasmania, three State agencies (Department of Primary Industries, Water and Environment, Department of Infrastructure, Energy and Resources, Department of Tourism, Parks, Heritage and the Arts) and three local councils (Kingborough Council, Brighton Council, Hobart City Council). While the design process involved the input of a comprehensive suite of perspectives, the opinions of drivers were not researched. As it is the behaviour of drivers that wildlife warning signs are attempting to influence, we suggest that drivers should be consulted as to what sign designs they would be more likely to respond.

Improving the design of wildlife warning signs to increase the likelihood of driver response has the potential to reduce the incidence of wildlife–vehicle collisions. To achieve this, several steps must be taken, the first of which is to examine and assess the opinions of drivers on wildlife warning sign designs through a comprehensive public opinion survey. In doing this, a range of sign designs should be provided alongside currently used signs, so that sign designs are presented equally and bias towards either current or alternative designs is avoided. More than one such public opinion survey study may need to be conducted in order to refine the sign designs. This is a crucial precursor to the process of assessing, approving, producing and experimentally field-testing those wildlife warning sign designs with the best likelihood of response and/or include features deemed as important to eliciting a response by drivers. The objective of the present study was to initiate this process and conduct the first public opinion survey on wildlife warning sign designs. More specifically, the project aimed to: (1) evaluate the importance of sign elements (*i.e.*, time period specifications and updated road-kill counts); (2) evaluate the importance of sign temporal and spatial placement; (3) design alternative wildlife warning signs; (4) evaluate and rank current and alternative sign designs by assessing the relative likelihood of driver response; (5) identify the messages conveyed by the top ranking signs; and (6) identify key features of the top ranking signs that may increase their noticeability, and therefore driver response.

## 2. Methods

### 2.1. Alternative Sign Designs

**Figure 1:**
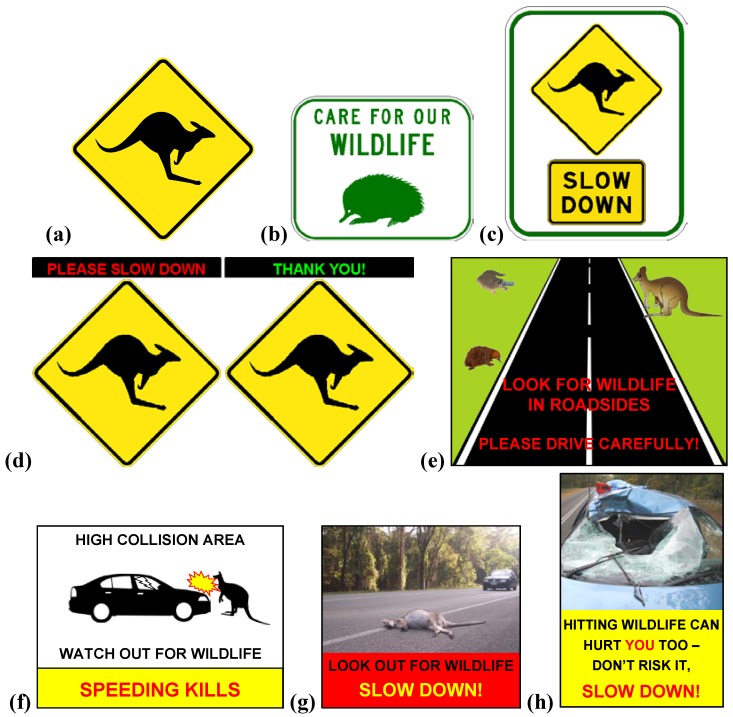
The eight signs included in the survey. (**a**) sign #1, W5-29, source: DTMR; (**b**) sign #2, TC1588, source: DTMR; (**c**) sign #3, TC1621, source: DTMR; (**d**) sign #4, W5-29 (modified), sign image source: DTMR; (**e**) sign #5, wildlife images source: Phillip Martin; (**f**) sign #6; (**g**) sign #6, photo source: A. Bond; (**h**) sign #8, photo source: Western Australia Police. The following note accompanied sign #4: “A single sign is displayed; the electronic message that is displayed above the sign is variable. The first message is activated when a speeding vehicle is detected and the second message is activated if the speeding vehicle slows down.”

There is a variety of currently used wildlife warning signs in Australia, the design of which does not vary greatly, likely due to standards set by respective road authorities, e.g., [16,17]. To our knowledge, there has been no attempt to assess the effectiveness of these standard designs or to use alternative designs. The most commonly used wildlife warning sign in Australia consists of a reflective yellow diamond with a black animal silhouette (e.g., Figure 1a). The wildlife signage guidelines for the Queensland Department of Transport and Main Roads [17] stipulate that this style of sign is only to be used for animals large enough to potentially cause human injury and/or vehicle damage. The koala (*Phascolarctos cinereus*) is one exception to this rule, as it is thought that drivers are likely to avoid hitting a koala, as it is an “endeared national symbol of Australia” [17], and in doing so potentially risk danger to themselves and/or occupants of other vehicles. For smaller animals that are unlikely to cause harm to the vehicle occupants and/or vehicle damage, wildlife information signs (e.g., Figure 1b) are used to inform of the presence of these animals. Due to the small potential for human injury and vehicle damage to result from collisions with small wildlife, some road agencies do not expect these signs to elicit a response from drivers [17]. High impact wildlife warning signs (e.g., Figure 1c) are very selectively used in areas where the risk of hitting an animal that may cause human injury and/or vehicle damage is significant [17].

In a collaborative effort to produce alternative wildlife warning sign designs, a group of ecologists from various backgrounds met on 14 March 2013 at Griffith University, Brisbane, and discussed possible features that may elicit greater responsiveness from drivers [18]. Some of the ideas produced from this meeting and used to design alternative sign designs are summarised in Table 1. 

**Table 1:** Ideas for alternative wildlife warning sign designs produced from the meeting held on 14 March 2013.

Sign design features
Signage with animal-activated flashing lights or electronic message
Vehicle speed-activated flashing lights or message “SLOW DOWN PLEASE”, and then, if the vehicle slows “THANK YOU”—positive reinforcement
Display cute images of wildlife to appeal to people’s sense of protection for animals
Take a more anthropocentric approach—display images or messages that allude to human injury and vehicle damage from colliding with large wildlife
Use more realistic and graphic images of wildlife–vehicle collisions (e.g., a car after hitting a kangaroo)
Display “HIGH WILDLIFE COLLISION ZONE” instead of “WILDLIFE” or “WILDLIFE ZONE”—more specifically descriptive of potential danger
Include words to encourage drivers to be more vigilant, e.g., “LOOK FOR WILDLIFE ON ROADSIDES”
Display the number of animals killed on that road over the previous year (or other time period)

A Google image search for “wildlife warning road signs” was also conducted to assist in designing signs that vary from those commonly used in Australia. Alternative signs were designed and selected by the researchers, as funds for a graphic designer were not available. 

### 2.2. Public Opinion Survey

The public opinion survey was primarily designed to enable the comparison of the likelihood of responses to each sign and determine the value of sign components. The sign component and placement questions discussed here were:

“Are you more or less likely to respond to a sign with specified time periods with increased alertness and reduced driving speed if you are driving during this period (6 pm–6 am, Aug–Dec)? e.g., 8 pm in November” (answer options: “more likely”, “less likely”, and “I am equally likely to respond to signs in this way, whether or not time specifications are displayed”); 

“Are you more or less likely to respond to a sign with specified time periods with increased alertness and reduced driving speed if you are driving outside this period (6 pm–6 am, Aug–Dec)? e.g., 10 am in April” (answer options: “more likely”, “less likely”, and “I am equally likely to respond to signs in this way, whether or not time specifications are displayed”); 

“Are you more or less likely to respond to a sign that displays the number of road-killed animals that have occurred on that road over the previous year with increased alertness and reduced driving speed?” (answer options: “more likely”, “less likely”, and “I am equally likely to respond to signs in this way, whether or not road-kill numbers are displayed”);

“Are you more likely to respond with increased alertness and reduced driving speed to a permanent, periodic or temporary wildlife warning sign?” (answer options: “permanent”, “periodic”, “temporary”, and “I am equally likely to respond to a sign whether it is permanent, periodic or temporary”); and 

“Are you more likely to notice a wildlife warning sign if it is positioned on the roadside or in the median strip (where available)?” (answer options: “roadside”, “median strip”, and “I am equally likely to notice both”).

Three standard wildlife warning signs (approved by the Department of Transport and Main Roads, Figure 1(a–c)) and five alternative signs (Figure 1(d–h)) were included in the survey. Each sign was graphically presented in the survey and the participants asked a series of questions. The main question used to compare sign designs was “Please indicate the likelihood that you would respond to this sign by increasing alertness and reducing driving speed when driving at the following speeds”. Here a five-part Likert-type scale (5 - highly likely, 4 - likely, 3 - unsure, 2 - unlikely, 1 - highly unlikely) was used to determine the likelihood of response to each sign at 60 km/h, 80 km/h and 100 km/h. This same question was asked separately regarding an animal-activated sign and a vehicle speed-activated sign, neither of which were displayed graphically. For each sign, participants were also asked “Please explain the message that is conveyed to you by this sign” and “What part or aspect of this sign stands out the most to you?” in open form answers. Smaller images of all the sign designs were then displayed together and participants were then asked “Of all of the signs displayed above, which one are you most likely to respond to by increasing alertness and reducing driving speed?”. In addition, participants were asked about their general driver experience, whether they had previously been involved in collisions with animals and their age, gender and state of residence. However participant demographics and experience are irrelevant when assessing driver response to wildlife warning signs, as signs are targeted at all drivers. Therefore, for the purposes of this study, responses to questions were not compared between participant demographics and experiences. This research was approved by the Griffith Human Research Ethics Committee on 3 June 2013 (ENV/18/13/HREC). The survey was delivered through the online survey tool SurveyMonkey. See supplementary material 1 for the complete survey. 

### 2.3. Participant Recruitment

The survey was aimed at persons 18 years or older who held a licence that enabled them to drive in Australia. Participants to the survey were recruited mainly by emailing and Facebook posts to existing networks of colleagues, associates, friends and family, and encouraging the forwarding on through subsequent networks. Although this method of recruitment was not random, it ensured that the survey was at least distributed to people with a range of backgrounds, education and values. A webpage dedicated to the survey was also created on the My Roadkill website (myroadkill.com.au) to assist in recruiting participants. The survey was also posted to the My Roadkill Facebook page. The Royal Automobile Club of Queensland (RACQ), the Royal Automobile Club of Victoria (RACV) and the National Roads and Motorists’ Association (NRMA) were approached to assist in focusing recruitment to drivers and to reach a broader audience, but all declined. RACV declined to participate because the alternative sign designs were not approved by any road agency; RACQ and NRMA did not give reasons for declining to participate. Due to the limited funds and time in which survey responses were required, the survey could not be advertised or distributed using any other methods.

From here onwards, the animal-activated and vehicle speed-activated signs will be referred to as the A-A and VS-A signs, respectively. Additionally, for conciseness, the terms ‘appropriate response’ and ‘to respond’ are used when referring to drivers increasing alertness and decreasing driving speed in response to signs.

### 2.4. Data Analyses

Separate Chi-square tests were conducted on the likelihood of participants to respond to signs displaying time specifications, both within and outside the specified time period, and to signs displaying the number of animals previously killed along the road. Chi-square tests were also conducted on the likelihood of participants to respond to permanent, periodic and temporary signs and signs placed on the roadside or median strip. Periodic signs were defined as being displayed for a two-week period every three months and temporary signs were defined as being displayed for a one month period every year.

Chi-square tests were conducted comparing participant answers to the question “Please indicate the likelihood that you would respond to this sign by increasing alertness and reducing driving speed when driving at the following speeds” for each of the signs (including the A-A and VS-A signs). Separate tests were conducted for participant answers at 60 km/h, 80 km/h and 100 km/h. To reduce the occurrence of values less than five in the contingency tables, the categorical answers of “highly likely” and “likely”, and “highly unlikely” and “unlikely” were pooled. This resulted in a 10 (signs) × 3 (answers) contingency table being analysed for each speed.

Additionally, the signs were ranked using the Likert-type scale responses to the above question by summing the scores for the three speed items for each participant, and then averaging these across all participants. This gave a minimum possible score of 3 and a maximum possible score of 15, with higher scores relating to higher likelihood of response. Signs were then ranked according to their mean score. One participant did not answer this question for sign #3 at all three speeds and was excluded from the calculations for this sign. Scored data from Likert-type scales are ordinal (unless distances between the scale options can be justified as equal), and as such parametric statistics and the use of means are inappropriate [19]. Here, however, mean sign scores were not compared directly; mean values were only obtained in order to rank the signs based on answers given on the Likert-type scale. Such ranking would not be possible using median or mode values.

Separate Chi-square tests were conducted to test for even distribution of participant answers to the two questions “Of all of the signs displayed above, which one are you most likely to respond to by increasing alertness and reducing driving speed?” and “Of all of the signs displayed above, which one do you think drivers in general are most likely to respond to by increasing alertness and reducing driving speed?”.

All statistical analyses were performed in the statistical program R [20]. 

## 3. Results

### 3.1. Public Survey Participants

A total of 134 complete survey responses were received. The majority of these participants held an Australian drivers’ licence (98.5%), resided in Queensland (70.1%), and were female (69.4%). A range of ages and length of driving experience of participants were included in the survey. Most participants drove daily (76.1%) or one to a few times per week (17.2%) and regularly drove in areas that displayed wildlife warning signs (69.4%). See supplementary material 2 for further details.

### 3.2. Sign Elements and Placement

**Figure 2:**
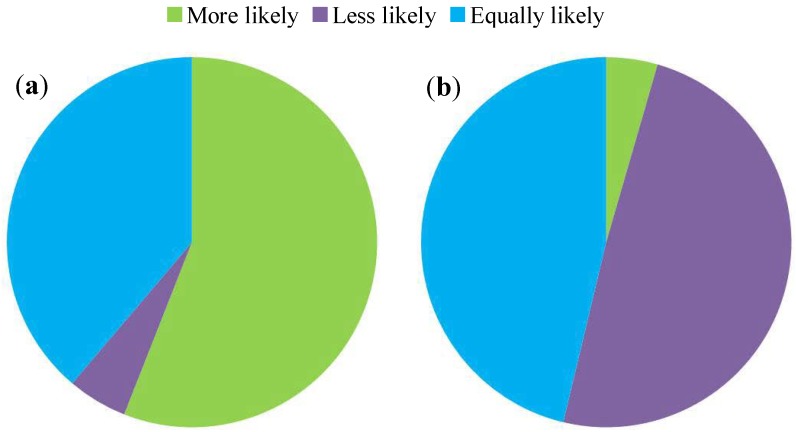
The proportion of participants that were more likely, less likely and equally likely to respond to a sign displaying specified time periods (**a**) inside and (**b**) outside the specified time period. N = 134.

**Figure 3:**
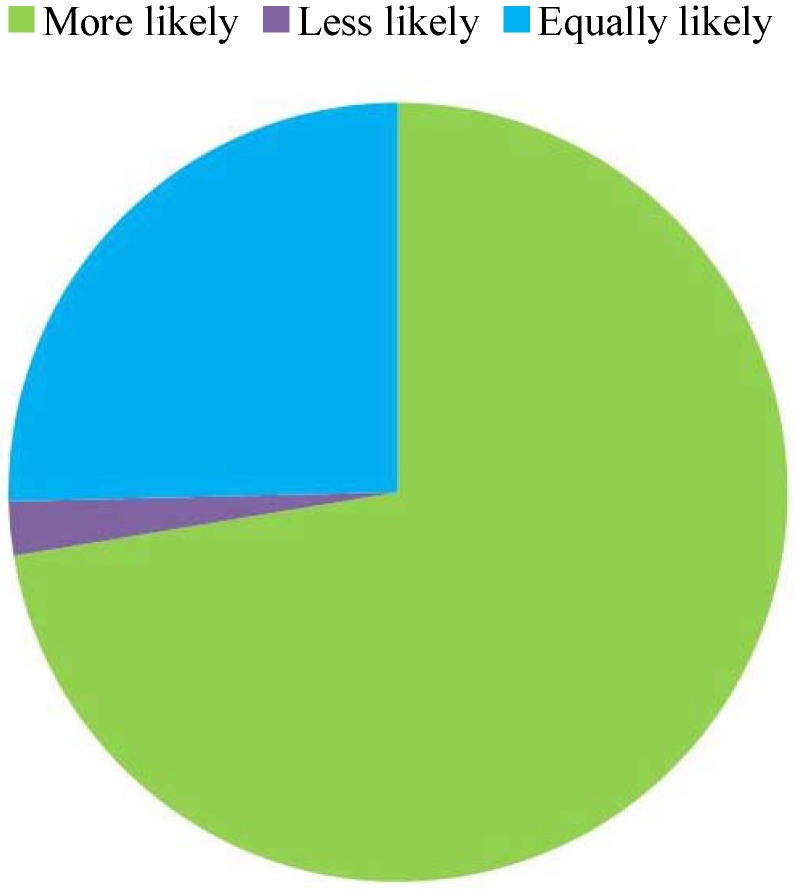
The proportion of participants that were more likely, less likely and equally likely to respond to a sign displaying the number of road-killed animals over the previous year (or other time period) for the section of road. N = 134.

The presence of season and time indicators on wildlife warning signs altered the likelihood that participants would react to signs inside and outside the specified periods. During the specified time period, 56.0% of participants were more likely, 38.8% were equally likely, and 5.2% were less likely to respond appropriately (χ^2^ = 53.567, df = 2, p = 2.334 × 10^−12^, Figure 2a). Outside the specified time period, 49.3% of participants were less likely, 46.3% were equally likely, and 4.5% were more likely to respond appropriately (χ^2^ = 50.388, df = 2, p = 1.144 × 10^−11^, Figure 2b). The addition of a count of the number of animals killed by vehicles over the previous year (or other time period) significantly increased the likelihood that participants would react to the wildlife warning sign (72.4% more likely to respond, χ^2^ = 102.731, df = 2, p = 2.2 × 10^−16^, Figure 3). 

When questioned about the temporal and spatial placement of signs, the majority of participants were equally likely to respond (55.2% and 59.7%, respectively), regardless of how frequently or where the sign was placed. Permanent, periodic and temporary placements of signs were more likely to produce a response in 23.1%, 13.4% and 8.2% of participants, respectively. The placement of signs on the roadside was more likely to produce a response in 31.3% of participants, whereas this was true for only 9.0% of participants for a sign placed in the median strip.

### 3.3. Sign Design Comparisons

**Figure 4:**
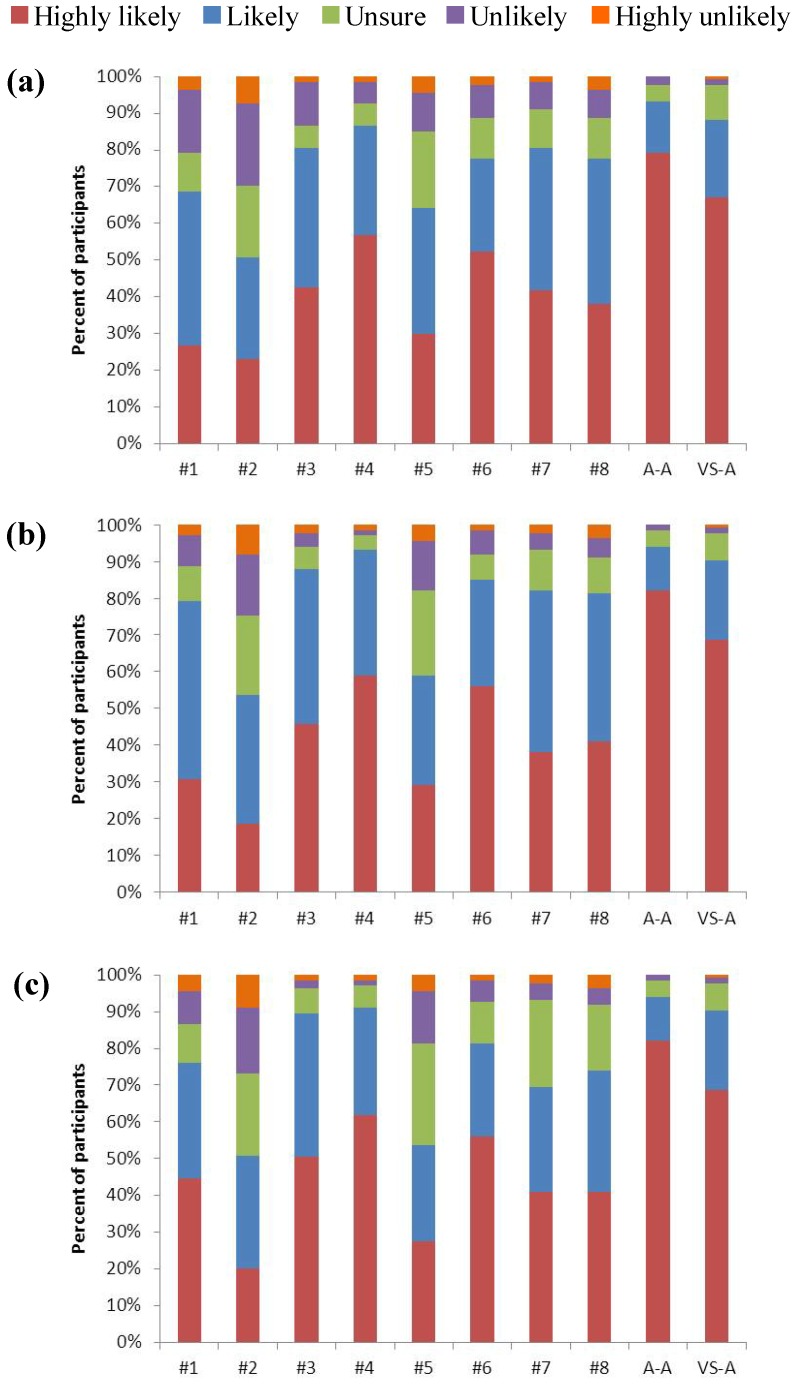
Participant likelihood to respond appropriately to the signs at (**a**) 60 km/h, (**b**) 80 km/h, and (**c**) 100 km/h. For sign #3 N = 133; for all other signs N = 134.

The likelihood that participants would respond to signs with reduced driving speed and increased vigilance was significantly different between the ten signs at all three speeds (60 km/h: χ^2^ = 126.105, df = 18, p < 2.2 × 10^−16^; 80 km/h: χ^2^ = 152.771, df = 18, p < 2.2 × 10^−16^; 100 km/h: χ^2^ = 187.429, df = 18, p < 2.2 × 10^−16^). At 60 km/h, the A-A sign, VS-A sign, sign #4, sign #3 and sign #7 were more likely to produce an appropriate response than the other signs (Figure 4a). At 80 km/h and 100 km/h, the A-A sign, VS-A sign, sign #4, sign #3 and sign #6 were more likely to produce an appropriate response than the other signs (Figure 4(b,c)). 

**Figure 5:**
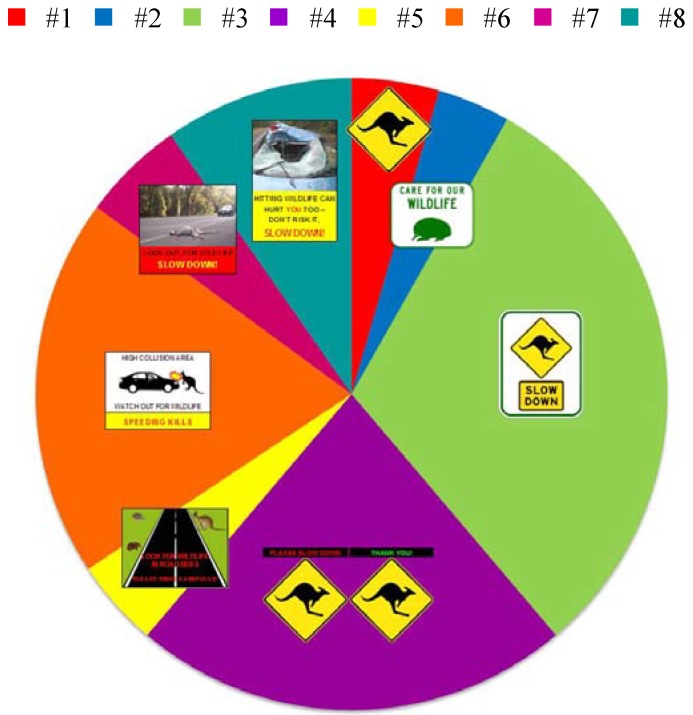
The proportion of participants that preferenced the signs as the most likely to produce the response of increased alertness and reduced driving speed. N = 134.

The sign most likely to produce an appropriate response from participants was unevenly distributed among the signs (χ^2^ = 79.254, df = 7, p = 1.955 × 10^−14^). Sign #3 (30.6%), sign #4 (22.4%) and sign #6 (19.4%) were the most frequently preferred signs by participants (Figure 5). 

Using the ranking from the mean scores (from the Likert-type scale) for each sign and the preference ranking, sign #3, sign #4 and sign #6 were the three highest ranked signs using both methods (Table 2). Despite this, the sign placement in the top three signs varied between the rankings; the scored and preference ranks align for only the three lowest ranked signs. When the A-A and VS-A signs were included in the scored rankings, they were the highest ranked signs, respectively. 

A variety of messages were conveyed to participants by the three highest ranking signs. The most consistently conveyed messages were for drivers to slow down, that there is a high animal presence in the area and that there is a crash risk (Table 3). Participants also mentioned numerous aspects of each sign as the most conspicuous with words relating to speed, images, the electronic message and colours being the elements most consistently highlighted (Table 4). It should also be noted that some participants stated that sign #6 and sign #8 were too cluttered and had too many words to be able to read at speed. 

**Table 2:** Sign mean scores (from the Likert-type scale) and ranks based on both scores and participant preferences (see Figure 5). The shaded rows represent the three highest ranking signs (not including the animal-activated (A-A) and vehicle speed-activated (VS-A) signs). For sign #3 N = 133; for all other signs N = 134. * The A-A and VS-A signs were ranked separately so that scored and preference ranks could be directly compared, as these signs were not included in the participant preference question. The scored ranks in parentheses are those when A-A and VS-A signs were included in the ranking.

Sign	Sign image	Mean score ± SE	Scored rank*	Preference rank
#1	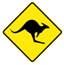	11.7 ± 0.24	6 (8)	6
#2	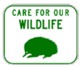	10.1 ± 0.30	8 (10)	8
#3	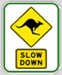	12.7 ± 0.21	3 (5)	1
#4	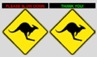	13.3 ± 0.20	1 (3)	2
#5	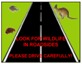	11.0 ± 0.27	7 (9)	7
#6	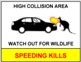	12.8 ± 0.24	2 (4)	3
#7	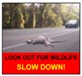	12.2 ± 0.23	4 (6)	5
#8	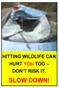	12.1 ± 0.25	5 (7)	4
A-A	-	14.2 ± 0.16	(1)	-
VS-A	-	13.6 ± 0.20	(2)	-

**Table 3:** The percent of participants to which various messages were conveyed by each of the three highest ranking signs. Note that multiple messages were conveyed to some participants, and so the percentages for each sign do not add up to 100%. N = 134 for each sign.

Message conveyed	Percent of participants		
	Sign #3	Sign #4	Sign #6
Slow down	63.4	58.2	25.4
High animal presence	57.5	29.9	22.4
Crash risk	21.6	10.4	41.8
Be careful/vigilant	23.9	10.4	17.9
Personal injury	-	-	27.6
Animal welfare	-	3.7	17.9
Thank you	-	14.2	-
Limit speed	-	2.2	1.5
Urgency	1.5	1.5	-
Speed enforcement	-	3	-
Important wildlife area	-	0.7	-
Unusable answer	1.5	6.7	5.2

**Table 4:** The percent of participants to which various sign features stood out in each of the three highest ranking signs. Note that multiple sign features stood out to some participants, and so the percentages for each sign do not add up to 100%. N = 134 for each sign.

Emphasised feature	Percent of participants		
	Sign #3	Sign #4	Sign #6
“Slow down” / “speeding kills”	73.1	10.4	30.6
Image	29.9	25.4	56
Electronic message	-	47.8	-
Colour	11.2	9	14.2
Yellow/black contrast	8.2	11.2	1.5
“High collision area”	-	-	11.9
Speed detection	-	9.7	-
“Thank you”	-	7.5	-
Words	-	3.7	1.5
Large size/shape	3.7	-	-
Multiple sign panels	1.5	-	-
“Watch out for wildlife”	-	-	0.7
Unusable answer	6	6.7	8.2

## 4. Discussion

### 4.1. Sign Elements and Placement

Almost three-quarters of participants said that they were more likely to respond to a wildlife warning sign that displayed an updated number of animals killed on the road over some preceding period of time. This was an important finding. The addition of this feature as a separate panel to existing wildlife warning signs would be relatively simple on roads where regular road-kill surveys are already conducted. This information feedback to drivers would provide them with evidence that wildlife–vehicle collisions do occur regularly in that location, especially where the bodies of road-killed animals are removed. 

In a series of experiments on methods to reduce driver speed, van Houten and Nau [21] found that a brief information feedback program was successful in reducing the proportion of drivers speeding. The program involved giving speeders a warning ticket and a flier on the numbers of collisions, injuries and fatalities that had occurred on that specific road in the past. Even though the program only ran for one to four days, it appeared to affect vehicle speeds for several weeks after its conclusion [21]. Some of the participants in the present study expressed that they were much less likely to respond to signs if there was no evidence that wildlife–vehicle collisions occur regularly. This unfortunately is hampered by the regular removal of animal carcasses from roads, usually by local councils, but also occasionally by members of the public [22]. The potential importance of road-kill being present has also been supported by studies assessing the effect of various wildlife warning signs in combination with animal decoys or carcasses at the roadside [9,23].

Some wildlife warning signs display specified time periods, e.g., Aug–Dec, 7 pm–5 am [13] during which drivers are to be aware of the risk of colliding with an animal. Such temporal indications of increased risk of wildlife–vehicle collisions are highly relevant in areas where animals cross roads at predictable times of the year, such as seasonally migratory deer [10,23] and amphibians [24,25] in North America and Europe. In Australia, however, most species that are commonly impacted by road mortality are not migratory, and thus are less predictable as to the timing of road crossings, e.g., [26,27]. In the current study, 56% of participants said that they were more likely to respond to a sign that displayed time specifications within the specified time period, although 49.3% of participants also indicated that they were less likely to respond outside the specified time period. This suggests that drivers think there is a much lower likelihood of encountering an animal on the road outside the specified time period, when there may be little difference. This may have the unwanted consequence of drivers being less vigilant for animals around the road during certain times of the year and potentially increasing wildlife–vehicle collisions. Additionally, a small number of participants (5.2%) answered that they were less likely to respond to such a sign within the specified time period, perhaps because they perceived that, if animals were only likely to be near the road during a certain time of the year, they were generally less likely to encounter an animal. The relevance and potential benefits and detriments of displaying seasonal specifications on wildlife warning signs need to be carefully considered when proposing to install signs in the future. It is recommended that such time specifications only be used in situations where there is definitive evidence of much higher road-kill rates during certain periods, and not when seasonal variations are only slight. This will ensure that drivers will continue to be vigilant and respond to signs throughout the year.

Wildlife warning signs are usually installed permanently, although in some locations, temporary signs are used when warning of migrating species, e.g., [10]. The use of these temporarily or periodically placed wildlife warning signs in these situations is appropriate where the period of high risk of wildlife–vehicle collisions is highly predictable. It is, however, possible that a periodic or temporary placement strategy may reduce driver habituation to signs, as this is a significant concern regarding the long-term effectiveness of wildlife warning signs [14]. When queried as to the temporal placement of signs that would more likely produce an appropriate response to wildlife warning signs, the majority of participants to the present study answered that permanent placement would be more effective, or that their response would be equal regardless of temporal placement. However, such a question may be very difficult to answer reliably, as participants may be unaware of whether they are likely to become habituated to signs, and thus their answer may not align with their actions when placed in the real situation. Some indication of participants’ awareness of the potential to habituate to signs is suggested by small proportions of participants answering with periodic and temporary placement. Therefore, field trials comparing the long-term effectiveness of permanent, periodic and temporary warning signs on driver speed and the occurrence of wildlife–vehicle collisions would be highly valuable.

The placement of signs on either the roadside or the median strip may impact on the noticeability of the signs and thus the likelihood that drivers will respond to them. Most wildlife warning signs are currently positioned on the roadside, although the median strip has also been used, e.g., [14], presumably where there are fewer obstructions to the visibility of the sign. The majority of participants indicated that they were equally likely to respond regardless of where the sign was positioned, whereas almost one-third of participants gave preference to the roadside and only 9% gave preference to the median strip. From these results, it would be advisable to only place wildlife warning signs in the median strip when clear visibility and/or noticeability of the sign on the roadside would be hampered by an obstruction or driver view may be distracted by other signage and/or objects at the roadside. 

Many wildlife warning signs also display the distance for which they are applicable. Hardy and colleagues [28] suggested that a shorter displayed distance may increase driver response to a portable dynamic message sign (displaying “next 2 miles”) compared to the permanent dynamic message signs (displaying “next 20 miles”). This aspect of wildlife warning signs was not addressed by the current study. It would, however, be important to experimentally test the influence of the length of the warning distance displayed on signs to reveal optimal displayed warning distances and therefore a recommended distance for repeated signs along extended areas of wildlife–vehicle collision risk.

### 4.2. Sign Designs

The A-A and VS-A signs had consistently high likelihoods of participants responding appropriately at all three speeds, most likely due to the interactive nature of the signs. This finding was supported by the responses to sign #4 also having consistently high likelihoods of response and the electronic message being the feature of this sign that stood out to most participants. Additionally, when assessing the effectiveness of vehicle-activated signs, Sullivan and colleagues [14] found that mean vehicle speed was lowest when the associated flashing lights were activated by vehicles traveling at 19 km/h (*i.e.*, the lights would have activated for most vehicles, not just speeding vehicles). It is likely that the A-A sign received slightly higher response rates due to the flashing light or message conveying that there is an immediate risk of collision with an animal. It is highly likely, however, that drivers would become less responsive to A-A signs if false triggers (for example, from moving vegetation) occurred frequently [29].

The most consistently conveyed messages associated with the signs used in the present study were for drivers to slow down, that there is high animal presence in the area, that there is a crash risk, and to be careful and/or vigilant. The message of potential human injury and/or vehicle damage was also important for the signs that conveyed this message. These messages should be incorporated into future wildlife warning sign designs as they are likely to be influential to driver behaviour. It is important, however, to keep the design of signs simple, perhaps with fewer words than some of the alternative sign designs used in this study. This would be particularly crucial along high speed roads, where drivers have less time to read and interpret signs. 

Wording relating to speed, images, and bright and contrasting colours appeared to attract the attention of participants, and are therefore likely to be important for maximising the noticeability of the sign. Where funds allow for electrical components, flashing lights and/or messages would additionally increase noticeability, even if such elements were not animal- or vehicle-activated. Even if the design of wildlife warning signs is enhanced, it is possible that some drivers will still become habituated to the signs and not always notice or respond to the signs. The incorporation of electronically-activated components in wildlife warning signs is likely to reduce the occurrence of habituation, as the electronic message and/or flashing lights would engage drivers and alert them to the potential risk. Even so, it is recommended that research into driver habituation to wildlife warning signs be conducted, through either repeated surveys that evaluate driver responses to the same signs over time, or long-term field trials on a variety of static and electronically-activated signs.

### 4.3. Study Concerns and Limitations

Due to the exploratory nature of this study, there are several issues that should be improved upon or explored in more depth in future research. This study primarily used kangaroo/wallaby related images in the sign designs to attempt to standardise the taxa to which drivers were responding, enabling the survey to focus more on design aspects, rather than the species/taxa displayed. Due to the sign type displayed as sign #2 was intended for use where small taxa may be hit, a kangaroo image was not available for this sign, and so an echidna image was used [17]. It should be noted that some drivers are likely to respond differently to different species/taxa displayed on wildlife warning signs [30]; however, this was not the focus of the current study. It would be highly informative for future research to examine the differences in driver response to varying taxa displayed on wildlife warning signs, so that in areas where several species/taxa are at risk of being hit by vehicles, the taxon that produces the greatest response from drivers should be displayed. It should also be noted that colour-blind drivers were not considered when designing the alternative signs used in the survey, and so the colour combinations used may not be appropriate for use as is. This should be investigated in future studies that explore alternative wildlife warning sign design. Additionally, the consultation and/or use of a graphics designer is recommended to assist in designing any alternative wildlife warning signs in the future.

When interpreting results from public opinion surveys, researchers must be mindful that participants may not always respond to some questions entirely truthfully (this may be unintentional), and there may be discrepancies between survey results and actual human behaviour [31,32]. Because of this, the main section of the survey used in the current study was designed to compare the relative responses of drivers to different signs, so that results are still indicative of true relative responses to the signs. Despite this, it is possible that participants rated the likelihood of their response to the sign that appeared first higher than what would be their actual behaviour, as it may have been perceived that this was the appropriate behaviour, and therefore the desired answer. On viewing subsequent signs, this perception may have changed, as participants realised the comparative nature of the survey [32,33]. Again, this may not have been intentional. This potential bias may have caused the discrepancies between sign scored and preference ranks. Such potential bias may have been avoided by showing the participants all signs used in the survey before asking questions about any one sign. Additionally, when asking participants of the likelihood of their response to the signs, no option was given for participants to respond by either increasing their alertness without reducing speed, or reducing speed without increasing alertness. These issues should be taken into consideration for any future research that involves similar relative comparisons of sign designs through public opinion surveys.

Generalised interpretations of the results from this study are also limited due to low sample size, dominance of Queensland participants and non-randomised recruitment of participants. Unfortunately due to the limited time available to recruit participants, lack of funds for advertising and unwillingness of RACQ, RACV and NRMA to participate in participant recruitment, these situations were unavoidable.

## 5. Conclusions

High road-kill rates that are experienced in some urban and peri-urban areas may be greatly reduced by optimising the effectiveness of wildlife warning signs, a relatively inexpensive mitigation measure. Additionally, due to the high frequency with which drivers in urban and peri-urban areas repeatedly drive along the same roads, driver habituation to wildlife warning signs may be greater than along highways more remote areas. Although this research is clearly also relevant to remote areas, repetitive driving along the same stretches of roads is unlikely to occur as regularly in such areas, and thus driver habituation may be less of an issue. Because of this potential for greater habituation in urban and peri-urban areas, use of a variety of sign designs, both spatially and temporally, may be valuable. Research into this possible effect is needed, however.

Much more research needs to be conducted into assessing current and alternative wildlife warning sign designs to optimise their effectiveness. None of the alternative signs designed in this study are recommended for field tests, but modifications to their design could be made and reassessed using a similar method. Eventually field tests should be conducted using optimally designed signs and currently used signs, as such applied research is likely to produce more definitive and realistic results. The addition of sign elements, such as seasonal and distance specifications and road-kill counts to wildlife warning signs should also be field tested, however, the use of seasonal specifications on wildlife warning signs is only recommended when highly relevant to the target species/taxa. This and further research into optimising the design of wildlife warning signs will hopefully contribute to reducing the frequency of wildlife–vehicle collisions, particularly in urban and peri-urban landscapes where such wildlife–human conflicts can be frequent. 

## Supplementary Files

Supplementary File 1
