# Supplementary material for: Wildlife Warning Signs: Public Assessment of Components, Placement and Designs to Optimise Driver Response
Source: Animals (Basel). 2013 Dec 17;3(4):1142–61. doi: 10.3390/ani3041142 (PMC4494358; doi:10.3390/ani3041142)
Supplement: Supplementary File 1 [file animals-03-01142-s001.docx]

### **Supplementary Material 1**

### Wildlife warning sign design survey

**
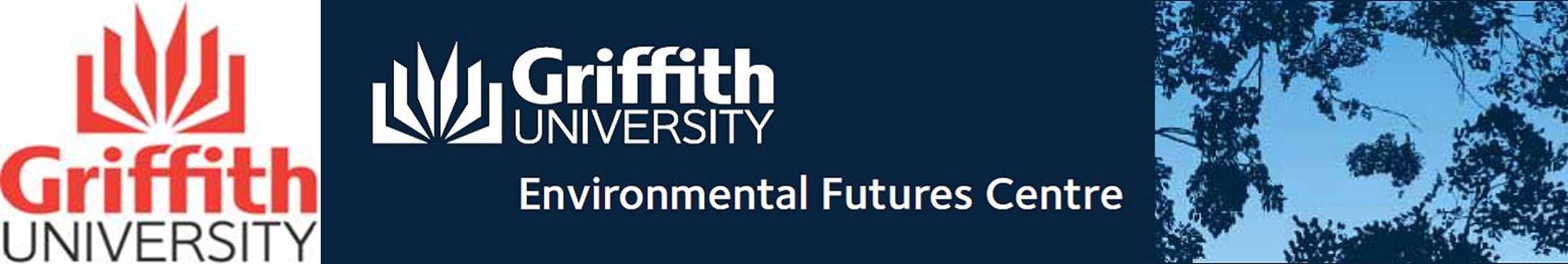
**

**Wildlife Warning Sign Design Survey Information Sheet**

This survey is part of a PhD research project.

This research project aims to provide potentially more effective wildlife warning sign designs by assessing the potential relative driver response to various signage designs through a public opinion survey.

Wildlife warning signs are commonly implemented mitigation measures to reduce wildlife-vehicle collisions in areas of high occurrence. Despite being the most commonly implemented road mitigation measure, evidence of their effectiveness in producing desired driver responses and reducing road-kill incidence is inconsistent and they are generally thought to not have an impact. Regardless of this, wildlife warning signs will continue to be implemented due to their inexpensive cost compared to other mitigation measures.

This research is part of a PhD research project and will contribute to a PhD thesis and scientific journal articles. This research is being conducted by PhD candidate Amy Bond ([A.Bond@griffith.edu.au](mailto:A.Bond@griffith.edu.au)) under the supervision of the chief investigator Associate Professor Darryl Jones ([D.Jones@griffith.edu.au](mailto:D.Jones@griffith.edu.au)). Please contact either researcher if you have any questions or concerns about this research. Participation in this survey is voluntary and you are able to exit the survey without submission at any point during the survey.

This survey is anonymous and your privacy is protected. At no point will your personal identity be asked or recorded.

Griffith University conducts research in accordance with the National Statement on Ethical Conduct in Human Research (2007). If you have any concerns or complaints about the ethical conduct of the research you should contact the Manager, Research Ethics on (07) 3735 4375 or [research-ethics@griffith.edu.au](mailto:research-ethics@griffith.edu.au).

Thank you for your interest and participation.

**
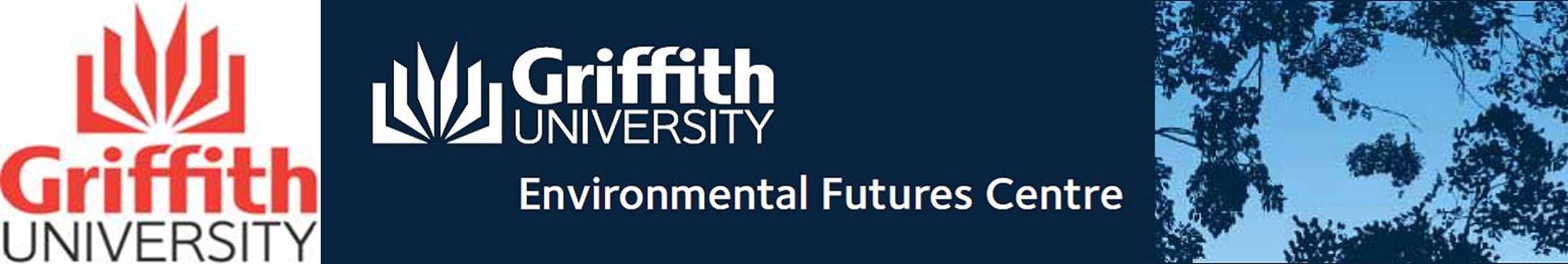
**

**Consent to Participate in the Wildlife Warning Sign Design Survey**

I confirm that I have read and understood the information provided and in particular I note that:

- my involvement in this research will be through a survey;
- I consent to the use of direct quotes;
- personal data will not be collected in any form;
- I have had all questions answered to my satisfaction;
- I understand the risks involved;
- there will be no direct benefit to me from my participation in this research;
- I will not be paid for my participation;
- my participation in this research is voluntary;
- I am free to withdraw from the survey at any time without comment or penalty;
- I can contact the student researcher, Amy Bond at [A.Bond@griffith.edu.au](mailto:A.Bond@griffith.edu.au), or the chief investigator, Associate Professor Darryl Jones at [D.Jones@griffith.edu.au](mailto:D.Jones@griffith.edu.au), if I have any questions or concerns about this research or to obtain a summary of the results of the research;
- I can contact the Manager, Research Ethics on (07) 3735 4375 or at [research-ethics@griffith.edu.au](mailto:research-ethics@griffith.edu.au) if I have any concerns about the ethical conduct of the project;
- I agree to participate in the project; and
- I am 18 years or older.

Completion and submission of this survey will be deemed to be consent to your participation in this research. Click next if you consent to participate in this survey.

**
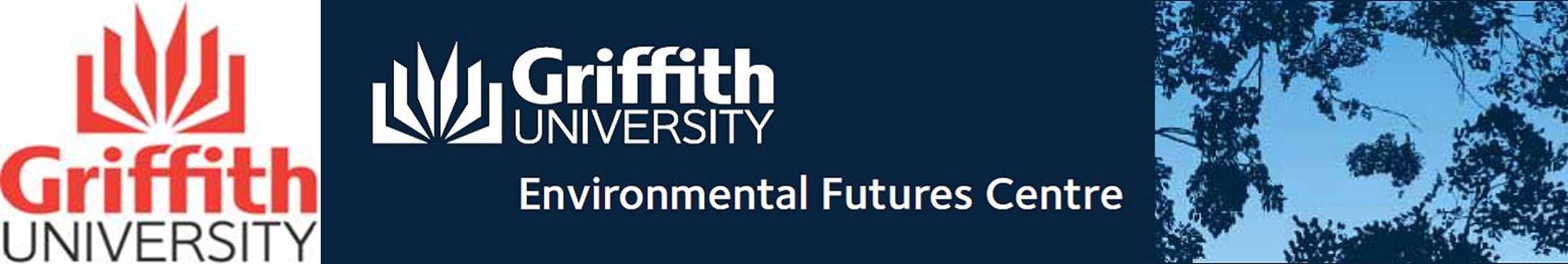
**

**Wildlife warning sign design survey**

**PART A**

1. Do you regularly drive in areas that display wildlife warning signs?
   1. Yes
   2. No
   3. Unsure
2. Have you ever hit a wild animal while driving? Please note that in this context a wild animal would be any animal that is not a domestic or stock animal and include both native and non-native species.
   1. Yes…please go to Q3
   2. No…please go to Q4
3. If you answered yes to Q2, how many wild animals do you estimate you have hit while driving?
   1. 1-5
   2. 5-10
   3. 10-30
   4. >30
4. Have you ever hit a domestic or stock animal while driving?
   1. Yes
   2. No
5. Do you usually notice wildlife warning signs?
   1. Yes
   2. No
   3. Sometimes (please explain)

_________________________________________________________________________________

_________________________________________________________________________________

1. When you see a wildlife warning road sign, what do you think is the desired driver response?
   1. Reduce driving speed
   2. Increase alertness
   3. Reduce driving speed and increase alertness
   4. Continue driving as before the sign
   5. Increase driving speed
   6. Other (please explain )

_________________________________________________________________________________

_________________________________________________________________________________

1. When you see a wildlife warning road sign, what is your usual response?
   1. Reduce driving speed
   2. Increase alertness
   3. Reduce driving speed and increase alertness
   4. Continue driving as before the sign
   5. Increase driving speed
   6. Other (please explain )

_________________________________________________________________________________

_________________________________________________________________________________

1. On wildlife warning signs, does the type of animal displayed make you more aware of that animal near the road? Please circle the most correct answer for you.
   1. Yes, I specifically look for the animal displayed
   2. Yes, I look for the animal displayed as well as other animals
   3. No, I look for any animals near the road
   4. No, I don’t actively look for animals near the road
2. Would you respond differently to different types of animals being displayed on wildlife warning signs? For example, would your response to a kangaroo warning sign be different to that of a koala, quoll, deer or wild boar?
   1. Yes
   2. No

Please explain:________________________________________________________________

_________________________________________________________________________________

_________________________________________________________________________________

1. On some roads, lesser-known animals (e.g. quolls or bandicoots) are the most likely animal to be involved in a collision with a vehicle. Does displaying an image of these animals on a sign (instead of the kangaroo) help your awareness of the animal of greatest concern?
   1. Yes
   2. No

Please explain why or why not:_____________________________________________

_________________________________________________________________________________

_________________________________________________________________________________

Some wildlife warning signs display specified time periods (e.g., 6pm – 6am, Aug – Dec). Are you more or less likely to respond to a sign with time periods with increased alertness and reduced driving speed if:

1. you are driving during this period (6pm – 6am, Aug – Dec)? e.g. 8pm in November
   - 1. More likely
     2. Less likely
     3. I am equally likely to respond to signs in this way, whether or not time specifications are displayed

1. you are driving outside this period (6pm – 6am, Aug – Dec)? e.g. 10am in April
2. More likely
3. Less likely
4. I am equally likely to respond to signs in this way, whether or not time specifications are displayed
5. Are you more or less likely to respond to a sign that displays the number of road-killed animals that have occurred on that road over the previous year with increased alertness and reduced driving speed?
   1. More likely
   2. Less likely
   3. I am equally likely to respond to signs in this way, whether or not road-kill numbers are displayed
6. Are you more likely to respond with increased alertness and reduced driving speed to a permanent, periodic or temporary wildlife warning sign?
   1. Permanent
   2. Periodic (sign is present for a two-week period every three months)
   3. Temporary (sign is displayed for a one month period every year)
   4. I am equally likely to respond to a sign whether it is permanent, periodic or temporary
7. Are you more likely to notice a wildlife warning sign if it is positioned on the roadside or in the median strip (where available)?
   1. Roadside
   2. Median strip
   3. I am equally likely to notice both

**PART B**

For the next set of questions, you will be shown several different wildlife warning signs and asked a set of questions about each sign. Please answer each question with regard to the sign displayed on that page only.

**Sign #1**

(Image source: Department of Transport and Main Roads)

1. Please explain how you are likely to respond to this sign if you saw it while driving.

_____________________________________________________________________________________________

_____________________________________________________________________________________________

_____________________________________________________________________________________________

_____________________________________________________________________________________________

1. Please explain the message that is conveyed to you by this sign.

_____________________________________________________________________________________________

_____________________________________________________________________________________________

_____________________________________________________________________________________________

_____________________________________________________________________________________________

1. Please indicate the likelihood that you would notice this sign when driving at the following speeds.

|  | Highly unlikely | Unlikely | Unsure | Likely | Highly  likely |
| --- | --- | --- | --- | --- | --- |
| 60 km/hr | 1 | 2 | 3 | 4 | 5 |
| 80 km/hr | 1 | 2 | 3 | 4 | 5 |
| 100 km/hr | 1 | 2 | 3 | 4 | 5 |

1. Please indicate the likelihood that you would respond to this sign by increasing alertness and reducing driving speed when driving at the following speeds.

|  | Highly unlikely | Unlikely | Unsure | Likely | Highly  likely |
| --- | --- | --- | --- | --- | --- |
| 60 km/hr | 1 | 2 | 3 | 4 | 5 |
| 80 km/hr | 1 | 2 | 3 | 4 | 5 |
| 100 km/hr | 1 | 2 | 3 | 4 | 5 |

1. What part or aspect of this sign stands out the most to you?

_____________________________________________________________________________________________

_____________________________________________________________________________________________

_____________________________________________________________________________________________

_____________________________________________________________________________________________

**Sign #2**

(Image source: Department of Transport and Main Roads)

1. Please explain how you are likely to respond to this sign if you saw it while driving.

_____________________________________________________________________________________________

_____________________________________________________________________________________________

_____________________________________________________________________________________________

_____________________________________________________________________________________________

1. Please explain the message that is conveyed to you by this sign.

_____________________________________________________________________________________________

_____________________________________________________________________________________________

_____________________________________________________________________________________________

_____________________________________________________________________________________________

1. Please indicate the likelihood that you would notice this sign when driving at the following speeds.

|  | Highly unlikely | Unlikely | Unsure | Likely | Highly  likely |
| --- | --- | --- | --- | --- | --- |
| 60 km/hr | 1 | 2 | 3 | 4 | 5 |
| 80 km/hr | 1 | 2 | 3 | 4 | 5 |
| 100 km/hr | 1 | 2 | 3 | 4 | 5 |

1. Please indicate the likelihood that you would respond to this sign by increasing alertness and reducing driving speed when driving at the following speeds.

|  | Highly unlikely | Unlikely | Unsure | Likely | Highly  likely |
| --- | --- | --- | --- | --- | --- |
| 60 km/hr | 1 | 2 | 3 | 4 | 5 |
| 80 km/hr | 1 | 2 | 3 | 4 | 5 |
| 100 km/hr | 1 | 2 | 3 | 4 | 5 |

1. What part or aspect of this sign stands out the most to you?

_____________________________________________________________________________________________

_____________________________________________________________________________________________

_____________________________________________________________________________________________

_____________________________________________________________________________________________

**Sign #3**

(Image source: Department of Transport and Main Roads)

1. Please explain how you are likely to respond to this sign if you saw it while driving.

_____________________________________________________________________________________________

_____________________________________________________________________________________________

_____________________________________________________________________________________________

_____________________________________________________________________________________________

1. Please explain the message that is conveyed to you by this sign.

_____________________________________________________________________________________________

_____________________________________________________________________________________________

_____________________________________________________________________________________________

_____________________________________________________________________________________________

1. Please indicate the likelihood that you would notice this sign when driving at the following speeds.

|  | Highly unlikely | Unlikely | Unsure | Likely | Highly  likely |
| --- | --- | --- | --- | --- | --- |
| 60 km/hr | 1 | 2 | 3 | 4 | 5 |
| 80 km/hr | 1 | 2 | 3 | 4 | 5 |
| 100 km/hr | 1 | 2 | 3 | 4 | 5 |

1. Please indicate the likelihood that you would respond to this sign by increasing alertness and reducing driving speed when driving at the following speeds.

|  | Highly unlikely | Unlikely | Unsure | Likely | Highly  likely |
| --- | --- | --- | --- | --- | --- |
| 60 km/hr | 1 | 2 | 3 | 4 | 5 |
| 80 km/hr | 1 | 2 | 3 | 4 | 5 |
| 100 km/hr | 1 | 2 | 3 | 4 | 5 |

1. What part or aspect of this sign stands out the most to you?

_____________________________________________________________________________________________

_____________________________________________________________________________________________

_____________________________________________________________________________________________

_____________________________________________________________________________________________

**Sign #4**

**PLEASE NOTE:** A single sign is displayed; the electronic message that is displayed above the sign is variable. The first message is activated when a speeding vehicle is detected and the second message is activated if the speeding vehicle slows down.

**PLEASE SLOW DOWN**

**THANK YOU!**

(Image source: Department of Transport and Main Roads)

1. Please explain how you are likely to respond to this sign if you saw it while driving.

_____________________________________________________________________________________________

_____________________________________________________________________________________________

_____________________________________________________________________________________________

_____________________________________________________________________________________________

1. Please explain the message that is conveyed to you by this sign.

_____________________________________________________________________________________________

_____________________________________________________________________________________________

_____________________________________________________________________________________________

_____________________________________________________________________________________________

1. Please indicate the likelihood that you would notice this sign when driving at the following speeds.

|  | Highly unlikely | Unlikely | Unsure | Likely | Highly  likely |
| --- | --- | --- | --- | --- | --- |
| 60 km/hr | 1 | 2 | 3 | 4 | 5 |
| 80 km/hr | 1 | 2 | 3 | 4 | 5 |
| 100 km/hr | 1 | 2 | 3 | 4 | 5 |

1. Please indicate the likelihood that you would respond to this sign by increasing alertness and reducing driving speed when driving at the following speeds.

|  | Highly unlikely | Unlikely | Unsure | Likely | Highly  likely |
| --- | --- | --- | --- | --- | --- |
| 60 km/hr | 1 | 2 | 3 | 4 | 5 |
| 80 km/hr | 1 | 2 | 3 | 4 | 5 |
| 100 km/hr | 1 | 2 | 3 | 4 | 5 |

1. What part or aspect of this sign stands out the most to you?

_____________________________________________________________________________________________

_____________________________________________________________________________________________

_____________________________________________________________________________________________

_____________________________________________________________________________________________

**Sign #5**

(Wildlife images source: Phillip Martin [phillipmartin.info])

1. Please explain how you are likely to respond to this sign if you saw it while driving.

_____________________________________________________________________________________________

_____________________________________________________________________________________________

_____________________________________________________________________________________________

_____________________________________________________________________________________________

1. Please explain the message that is conveyed to you by this sign.

_____________________________________________________________________________________________

_____________________________________________________________________________________________

_____________________________________________________________________________________________

_____________________________________________________________________________________________

1. Please indicate the likelihood that you would notice this sign when driving at the following speeds.

|  | Highly unlikely | Unlikely | Unsure | Likely | Highly  likely |
| --- | --- | --- | --- | --- | --- |
| 60 km/hr | 1 | 2 | 3 | 4 | 5 |
| 80 km/hr | 1 | 2 | 3 | 4 | 5 |
| 100 km/hr | 1 | 2 | 3 | 4 | 5 |

1. Please indicate the likelihood that you would respond to this sign by increasing alertness and reducing driving speed when driving at the following speeds.

|  | Highly unlikely | Unlikely | Unsure | Likely | Highly  likely |
| --- | --- | --- | --- | --- | --- |
| 60 km/hr | 1 | 2 | 3 | 4 | 5 |
| 80 km/hr | 1 | 2 | 3 | 4 | 5 |
| 100 km/hr | 1 | 2 | 3 | 4 | 5 |

1. What part or aspect of this sign stands out the most to you?

_____________________________________________________________________________________________

_____________________________________________________________________________________________

_____________________________________________________________________________________________

_____________________________________________________________________________________________

**Sign #6**

1. Please explain how you are likely to respond to this sign if you saw it while driving.

_____________________________________________________________________________________________

_____________________________________________________________________________________________

_____________________________________________________________________________________________

_____________________________________________________________________________________________

1. Please explain the message that is conveyed to you by this sign.

_____________________________________________________________________________________________

_____________________________________________________________________________________________

_____________________________________________________________________________________________

_____________________________________________________________________________________________

1. Please indicate the likelihood that you would notice this sign when driving at the following speeds.

|  | Highly unlikely | Unlikely | Unsure | Likely | Highly  likely |
| --- | --- | --- | --- | --- | --- |
| 60 km/hr | 1 | 2 | 3 | 4 | 5 |
| 80 km/hr | 1 | 2 | 3 | 4 | 5 |
| 100 km/hr | 1 | 2 | 3 | 4 | 5 |

1. Please indicate the likelihood that you would respond to this sign by increasing alertness and reducing driving speed when driving at the following speeds.

|  | Highly unlikely | Unlikely | Unsure | Likely | Highly  likely |
| --- | --- | --- | --- | --- | --- |
| 60 km/hr | 1 | 2 | 3 | 4 | 5 |
| 80 km/hr | 1 | 2 | 3 | 4 | 5 |
| 100 km/hr | 1 | 2 | 3 | 4 | 5 |

1. What part or aspect of this sign stands out the most to you?

_____________________________________________________________________________________________

_____________________________________________________________________________________________

_____________________________________________________________________________________________

_____________________________________________________________________________________________

**Sign #7**

(Photo source: Amy Bond)

1. Please explain how you are likely to respond to this sign if you saw it while driving.

_____________________________________________________________________________________________

_____________________________________________________________________________________________

_____________________________________________________________________________________________

_____________________________________________________________________________________________

1. Please explain the message that is conveyed to you by this sign.

_____________________________________________________________________________________________

_____________________________________________________________________________________________

_____________________________________________________________________________________________

_____________________________________________________________________________________________

1. Please indicate the likelihood that you would notice this sign when driving at the following speeds.

|  | Highly unlikely | Unlikely | Unsure | Likely | Highly  likely |
| --- | --- | --- | --- | --- | --- |
| 60 km/hr | 1 | 2 | 3 | 4 | 5 |
| 80 km/hr | 1 | 2 | 3 | 4 | 5 |
| 100 km/hr | 1 | 2 | 3 | 4 | 5 |

1. Please indicate the likelihood that you would respond to this sign by increasing alertness and reducing driving speed when driving at the following speeds.

|  | Highly unlikely | Unlikely | Unsure | Likely | Highly  likely |
| --- | --- | --- | --- | --- | --- |
| 60 km/hr | 1 | 2 | 3 | 4 | 5 |
| 80 km/hr | 1 | 2 | 3 | 4 | 5 |
| 100 km/hr | 1 | 2 | 3 | 4 | 5 |

1. What part or aspect of this sign stands out the most to you?

_____________________________________________________________________________________________

_____________________________________________________________________________________________

_____________________________________________________________________________________________

_____________________________________________________________________________________________

**Sign #8**

(Photo source: Western Australia Police)

1. Please explain how you are likely to respond to this sign if you saw it while driving.

_____________________________________________________________________________________________

_____________________________________________________________________________________________

_____________________________________________________________________________________________

_____________________________________________________________________________________________

1. Please explain the message that is conveyed to you by this sign.

_____________________________________________________________________________________________

_____________________________________________________________________________________________

_____________________________________________________________________________________________

_____________________________________________________________________________________________

1. Please indicate the likelihood that you would notice this sign when driving at the following speeds.

|  | Highly unlikely | Unlikely | Unsure | Likely | Highly  likely |
| --- | --- | --- | --- | --- | --- |
| 60 km/hr | 1 | 2 | 3 | 4 | 5 |
| 80 km/hr | 1 | 2 | 3 | 4 | 5 |
| 100 km/hr | 1 | 2 | 3 | 4 | 5 |

1. Please indicate the likelihood that you would respond to this sign by increasing alertness and reducing driving speed when driving at the following speeds.

|  | Highly unlikely | Unlikely | Unsure | Likely | Highly  likely |
| --- | --- | --- | --- | --- | --- |
| 60 km/hr | 1 | 2 | 3 | 4 | 5 |
| 80 km/hr | 1 | 2 | 3 | 4 | 5 |
| 100 km/hr | 1 | 2 | 3 | 4 | 5 |

1. What part or aspect of this sign stands out the most to you?

_____________________________________________________________________________________________

_____________________________________________________________________________________________

_____________________________________________________________________________________________

_____________________________________________________________________________________________

**PART C**

| **Sign #1** | **Sign #2** | **Sign #3** |
| --- | --- | --- |
|  | 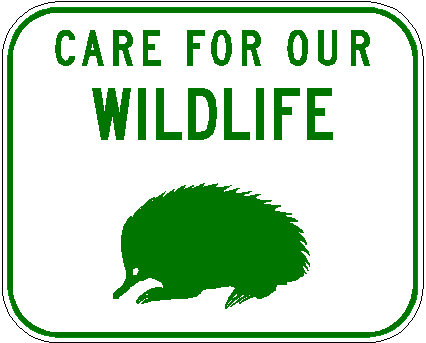 | 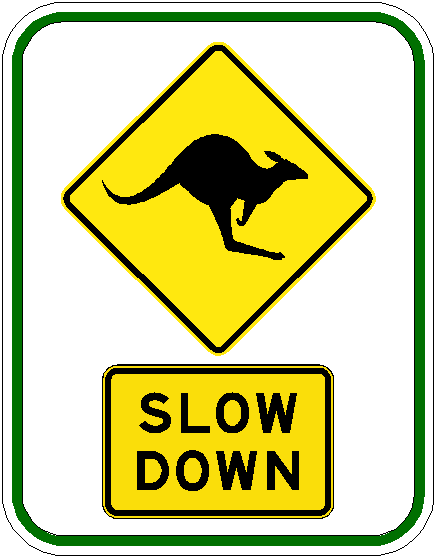 |
| Source: DTMR | Source: DTMR | Source: DTMR |

| **Sign #4**  ** ** | **Sign #5**  **** |
| --- | --- |
|  |  |
| Source: DTMR | Wildlife images source: Phillip Martin |

| **Sign #6** | **Sign #7** | **Sign #8** |
| --- | --- | --- |
|  |  |  |
|  | Photo source: Amy Bond | Photo source: WA Police |

1. Of all of the signs displayed above, which one are you most likely to respond to by increasing alertness and reducing driving speed?
   1. Sign #1
   2. Sign #2
   3. Sign #3
   4. Sign #4
   5. Sign #5
   6. Sign #6
   7. Sign #7
   8. Sign #8

Please explain why:__________________________________________________________

_________________________________________________________________________________

_________________________________________________________________________________

1. Of all of the signs displayed above, which one do you think drivers in general are most likely to respond to by increasing alertness and reducing driving speed?
2. Sign #1
3. Sign #2
4. Sign #3
5. Sign #4
6. Sign #5
7. Sign #6
8. Sign #7
9. Sign #8

Please explain why:__________________________________________________________

_________________________________________________________________________________

_________________________________________________________________________________

1. Please indicate the likelihood that you would respond to any sign that included flashing lights or an electronically displayed message that was activated by the presence of an animal at the roadside by increasing alertness and reducing driving speed when driving at the following speeds.

|  | Highly unlikely | Unlikely | Unsure | Likely | Highly  likely |
| --- | --- | --- | --- | --- | --- |
| 60 km/hr | 1 | 2 | 3 | 4 | 5 |
| 80 km/hr | 1 | 2 | 3 | 4 | 5 |
| 100 km/hr | 1 | 2 | 3 | 4 | 5 |

1. Please indicate the likelihood that you would respond to any sign that included flashing lights or an electronically displayed message that was activated by a speeding vehicle by increasing alertness and reducing driving speed when driving at the following speeds.

|  | Highly unlikely | Unlikely | Unsure | Likely | Highly  likely |
| --- | --- | --- | --- | --- | --- |
| 60 km/hr | 1 | 2 | 3 | 4 | 5 |
| 80 km/hr | 1 | 2 | 3 | 4 | 5 |
| 100 km/hr | 1 | 2 | 3 | 4 | 5 |

1. Do you have any additional comments about designing more effective wildlife warning signs?

_____________________________________________________________________________________________

_____________________________________________________________________________________________

_____________________________________________________________________________________________

_____________________________________________________________________________________________

_____________________________________________________________________________________________

_____________________________________________________________________________________________

_____________________________________________________________________________________________

**PART D**

1. Please indicate your age group.
2. 18-25
3. 25-40
4. 40-60
5. > 60
6. Please indicate your gender.
   1. Male
   2. Female
7. Where do you live?
8. QLD
9. NSW
10. VIC
11. TAS
12. SA
13. WA
14. NT
15. ACT
16. Outside Australia (please specify) ___________________________________
17. Do you currently have a driving licence?
18. Yes, I have an Australian licence
19. Yes, I have an international licence or a licence from another country that allows me to drive in Australia
20. No
21. How long have you had a driving licence?
22. < 1 year
23. 1-5 years
24. 5-10 years
25. 10-20 years
26. 20-40 years
27. > 40 years
28. How frequently do you drive?
29. Daily
30. One to a few times per week
31. One to a few times per month
32. Less than once a month

**Thank you** for taking the time to participate in this survey. Your answers are highly valuable and will hopefully contribute to designing more effective wildlife warning signs.

If you have any questions regarding this research, please contact Amy Bond by email at A.Bond@griffith.edu.au

### **Supplementary Material 2**

### Demographics and driving experience of respondents

**Table S1.** Survey respondent state of residence.

| **State of residence** | **Response percent** | **Response count** |
| --- | --- | --- |
| Queensland | 70.1% | 94 |
| New South Wales | 10.4% | 14 |
| Victoria | 3.0% | 4 |
| Tasmania | 0.7% | 1 |
| South Australia | 3.7% | 5 |
| Western Australia | 9.0% | 12 |
| Northern Territory | 0.7% | 1 |
| Australian Capital Territory | 2.2% | 3 |
| TOTAL | 100.0% | 134 |

**Table S2.** Survey respondent age.

| **Age** | **Response percent** | **Response count** |
| --- | --- | --- |
| 18-25 | 9.7% | 13 |
| 25-40 | 38.1% | 51 |
| 40-60 | 26.1% | 35 |
| > 60 | 26.1% | 35 |
| TOTAL | 100.0% | 134 |

**Table S3.** Survey respondent length of driving experience.

| **Years retained a drivers’ licence** | **Response percent** | **Response count** |
| --- | --- | --- |
| < 1 year | 2.2% | 3 |
| 1-5 years | 10.4% | 14 |
| 5-10 years | 14.2% | 19 |
| 10-20 years | 20.1% | 27 |
| 20-40 years | 24.6% | 33 |
| > 40 years | 28.4% | 38 |
| TOTAL | 100.0% | 134 |

**Table S4.** Survey respondent driving frequency.

| **Driving frequency** | **Response percent** | **Response count** |
| --- | --- | --- |
| Daily | 76.1% | 102 |
| One to a few times per week | 17.2% | 23 |
| One to a few times per month | 3.7% | 5 |
| Less than once a month | 3.0% | 4 |
| TOTAL | 100.0% | 134 |

**Table S5.** Survey respondent exposure to wildlife warning signs.

| **Do you regularly drive in areas that display wildlife warning signs?** | **Response percent** | **Response count** |
| --- | --- | --- |
| Yes | 69.4% | 93 |
| No | 27.6% | 37 |
| Unsure | 3.0% | 4 |
| TOTAL | 100.0% | 134 |
